# Anti-Inflammatory and Restorative Effects of Olives in Topical Application

**DOI:** 10.1155/2021/9927976

**Published:** 2021-06-26

**Authors:** Mahdiyeh Taheri, Leila Amiri-Farahani

**Affiliations:** ^1^Department of Reproductive Health and Midwifery, Faculty of Nursing and Midwifery, Iran University of Medical Sciences, Tehran, Iran; ^2^Department of Reproductive Health and Midwifery, Nursing Care Research Center, School of Nursing and Midwifery, Iran University of Medical Sciences, Tehran, Iran

## Abstract

**Methods:**

A literature search was conducted (1990–2021) in Medline, Embase, CINAHL, Google Scholar, Science Direct, SID, IranDoc, and Magiran databases. From the 102 reviewed articles, 17 articles were selected to be included in the current article.

**Results:**

Various forms of olive have long been used to accelerate the healing of various wounds and skin damage such as diabetic foot ulcers, atopic dermatitis, diaper dermatitis, episiotomy wound, and nipple ulcer but there are still no credible documents or articles that provide reliable evidence of topical use.

**Conclusion:**

According to the information obtained from the articles reviewed, olive oil appears to be an effective, safe, and available treatment. This study suggests that olive oil is an alternative remedy to minimize the frequent use of chemical-based treatments. More research may be beneficial to reach certainty in terms of curative properties of olive oil in similar or different injuries in different populations.

## 1. Introduction

Disruption in the integrity of skin, mucosal surfaces, or limb tissue can lead to wound formation. Wounds can occur as part of a disease process, either accidentally or intentionally [1]. In the healing process, several cellular and extracellular pathways are activated in a fully regulated and coordinated manner, with the aim of restoring tissue integrity. Classically, the wound healing process is divided into four distinct stages, including homeostasis, inflammation, proliferation, and regeneration of tissue. Delayed wound healing can be associated with higher morbidity and mortality of patients as well as appearance problems after the wound healing. It is estimated that the annual cost of wound healing complications in the United States alone is more than $ 1 billion [2]. Among the various compounds used for wound healing, we can refer to medicinal plants such as olives. The evergreen olive tree (Olea europaea) is a natural source of antioxidants and other bioactive compounds that come from different parts of the olive [3].

The effectiveness of olives on wound healing has been investigated in several studies [4–12]. In general, olives have antioxidant, antibacterial, anti-inflammatory [13], and antiviral properties and, therefore, can facilitate the repair of epithelial tissue, which is effective in the wound healing process [14]. A wide range of studies has shown that the phenolic compounds in olive ointment have anti-inflammatory effects, protective effects on neurons, antiaging effects, and cell repair properties [15–17].

Today, due to the increase in resistance of bacteria to antibiotics and the high cost of medical care, more attention has been paid to traditional therapies [18]. Since most chemical drugs contain preservatives, they can have negative effects on wound healing [19]. So far, many clinical trials have been conducted to investigate the effects of topical use of various olive products on wound healing, and in this article, we review the results of such studies. The purpose of this study is to collect the results of related studies, review them, and provide an abstract on the effects of olives on wound healing.

## 2. Methods

### 2.1. Design

This literature review article was completed following the academic standards for conducting integrative literature reviews [20]. We also as an additional element used Preferred Reporting Items for Systematic Reviews and Meta-Analyses (PRISMA) to structure the study and ensure the quality of the articles [21, 22]. The studies were included if they were interventional or pilot studies. We followed the methods of Leila Amiri-Farahani et al. [23].

### 2.2. Setting

Journal articles were examined in PubMed, CINAHL, PsycINFO, Web of Science, Ovid, Google Scholar, Science Direct, Cochrane Library, Magiran, Irandoc, and SID. The search protocol was based on the keywords: “olive,” “olive oil,” “topical application,” “wound,” “wound healing,” “ulcer,” and “sore.” These articles were peer-reviewed and published from 1990 to 2021.

### 2.3. Sample

**Figure 1:**
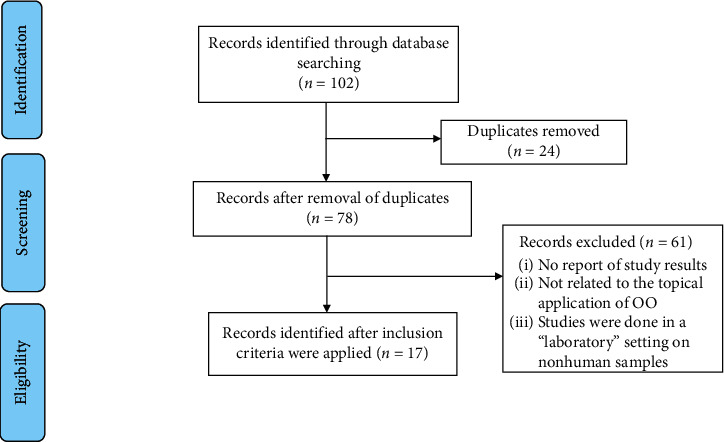
The articles included in the study.

Related articles were found in three steps. Initially, 102 articles were obtained using the aforementioned keywords. These results were then screened using exclusionary criteria. A total of 24 articles were excluded as they were duplicated. Exclusion criteria were [1] nonhuman samples and [2] protocol-based articles with no reported results. A total of 61 articles were excluded, leaving 17 articles for review (Figure 1).

### 2.4. Measurement

The author (MT) appraised each of the 17 articles which were peer-reviewed by another author (LAF) for accuracy. The extracted data included the title, country and city, participants' characteristics, intervention description, control or/and comparison groups, length of follow-up, the measure of outcome variables, and main results (Table 1).

## 3. Results and Discussion

Olive oil (OO) has different anti-inflammatory and restorative properties that may explain the effectiveness of OO when applied topically.

### 3.1. Oleic Acid

The OO is composed of approximately 98–99% fatty acids, mainly triacylglycerol, oleic acid esters (55–83%), palmitic acid (20–7.5%), linoleic acid (3.5%−21%), and other fatty acids such as stearic acid (0.5–5%) [35]. Oleic acid in olives can replace linoleic acid, and it is assumed that the main mechanism of action of linoleic acid is to modulate inflammation and stimulate skin regeneration [36]. Inflammation may also be the main process of linoleic acid activity, as it is a precursor of arachidonic acid. Arachidonic acid is metabolized to prostaglandins, thromboxanes, and leukotrienes, which promote local angiogenesis, fibroblast migration and differentiation, and extracellular matrix regeneration, all of which ultimately accelerate the wound healing process [37].

### 3.2. Olive Oil Phenolic Compounds (OOPCs)

The OOPC is currently believed to be involved in positive EVOO-related activities [38, 39]. In fact, the soluble part of olive oil is mainly made of OOPC, including phenolic acids, phenolic alcohols (hydroxyl tyrosols and tyrosols), secoiridoids such as oleuropein, hydroxytyrosol attached to the dialdehydic form of oleanolic acid, and flavonoids [40].

The phenolic compounds in olives (in topical use) have anti-inflammatory effects, and the polyphenols of olive oil are associated with neuroprotective and antiaging effect, so they can lead to the repair of epithelialized tissue that is effective in the wound healing process [41]. Also, the squalene compounds in olive oil include vitamins K, *D*, E, beta-carotene, and ubiquinol 10, which have antioxidant properties [42].

Studies have also shown that olive oil, when applied topically, leads to angiogenesis by increasing the levels of intravascular endothelial growth factor (VEGF) [43] and, with omega-3 fatty acids, can chronically inhibit the inflammation [44]. Also, extra virgin olive oil improves cell viability by increasing the capacity of antioxidants and providing higher MMP (mitochondrial membrane potential), which is essential for maintaining the mitochondrial function of keratinocytes [45]. Olives can facilitate wound healing by increasing epithelial regeneration [46].

## 4. Topical Applications of Olive

### 4.1. Nipple Sore

Nipple irritation is one of the most common complications in breastfeeding women. It was reported that 96% of mothers tend to not lactate due to nipple pain and ulcer during breastfeeding [47]. Since nipple-related damages and subsequent pains are important factors in the mother's decision to stop lactation, choosing the appropriate intervention is a dire need.

Oguz et al. conducted a study on 56 participants to determine the effectiveness of olive oil in the prevention of breast ulcers. At the end of the study, 89.2% of patients were more satisfied with the use of olive oil compared to lanolin (10.8%). They also found that ease of use and effectiveness were significantly higher in the OO group (*P* < 0.05), and also, 66.1% of the patients did not report pain in the breast after using olive oil compared to 46.4% who used lanolin. Also, no significant side effects were reported from the products [11]. In another study, Eshghizadeh et al. compared the effects of olive oil, aloe vera extract, and breast milk on improving breast cleft. At the end of the intervention, there was a significant difference (*P* < 0.05) between the three groups, so that in the AV group, the severity of breast cleft was the lowest. Improvement of breast cleft severity from the first to the seventh day in the AV group showed a greater decrease than the other two groups, and also a smaller decrease was observed in the OO group compared to the other two groups. There was a statistically significant difference between OO and AV groups (*P* < 0.003) in that regard [24].

### 4.2. Pressure Ulcer (PU)

PU is defined as necrosis of a part of the skin. This type of wound is caused by long-term pressure on the soft tissue between a prominent part of the bone and an external surface [48]. Numerous studies have examined the effect of olives on the prevention and improvement of PU. Varaei et al. conducted a study to compare the effect of massage with olive oil (OO) and sweet almond oil (SAO) on the prevention of pressure ulcers. The results showed that the incidence of pressure ulcers in the OO and SAO groups was lower than the control group, and also it was lower in the OO group than the SAO group (*P* < 0.05) [8]. In a study conducted by Poursadra et al., to compare the effect of massage with OO and henna oil (HO) on grade 1 pressure ulcers, a statistically significant difference was found between HO and OO (*P* < 0.05) in terms of the total score of the PUSH criterion. Therefore, they argued that HO and OO were both effective in healing pressure ulcers [25]. In a study conducted by Miraj et al., to determine the effect of olive oil on grade 1 pressure ulcers in ICU patients, a statistically significant difference was found in the total PUSH score between the two groups and the intragroup comparison of wound score in the OO group before and after the intervention (*P* < 0.05), while in the intragroup comparison in the control group, no change was observed [26]. Díaz-Valenzuela et al. investigated the effect of olives on the prevention of pressure ulcers in 571 patients with pressure ulcers. The results showed that the incidence of pressure ulcers in the olive oil solution group was 4.18% and in the HOFA group was 6.57% [27]. In a clinical trial, Bajwa et al. examined the effect of olive oil on the prevention of bed ulcer in 60 patients admitted to the ICU, and the results showed that the incidence of bed ulcer was significantly different between the two groups (*P*=0.03), so that the incident of bed ulcer in the OO group was significantly lower than the control group [28]. Findings from several studies indicate that olive oil, in addition to emollient effects on the skin, can be used topically in the treatment of skin problems such as psoriasis [49]. The mechanism by which the virgin olive oil exerts its protective effects on pressure wounds is not yet known, but it seems that compounds such as polyphenols and oleocanthal can lead to wound healing due to their anti-inflammatory properties and their positive effects on improving blood flow [50, 51].

### 4.3. Chronic Ulcers

In a pilot study, Vitsos et al. examined the effect of *Ceratothoa oestroides* olive oil extract in people with chronic ulcers. Treatment was evaluated using the Bates-Jensen scale, and the results showed that the overall wound score decreased by 36% (*P* < 0.001) [9].

### 4.4. Diabetic Wounds

Diabetic foot ulcer (DFU) is one of the most common and devastating complications of Diabetes mellitus (DM), which has indicated an increasing trend in the past decades [52]. Recent investigations have shown that more than 15% of patients with DM had DFU during their lifetime [53].

Nasiri et al., in a study, examined the effect of olive oil on diabetic foot ulcers in patients with type II diabetes and the results showed that OO significantly reduced the wound surface and depth and increased the general condition of the wound compared to the control group (*P* < 0.05) [6]. In the study of Karimi et al., to investigate the effect of olive oil and honey on the healing of diabetic foot ulcers in comparison with the control group, both olive oil and honey groups received a significantly better score in terms of the wound surrounding tissue, wound grade, wound discharge, and wound healing score than the control group (*P* < 0.05). The results of this study showed that olive oil and honey can be equally effective in wound healing [29].

### 4.5. Perineal Ulcer

The perineal inflammations lead to the mother's pain and discomfort and consequently reduced the ability to take care of herself, the baby, and the family. Studies also have indicated the septic shock and death in long-term infection of the perineum [54].

Amani et al., in a study, compared the effect of cold gel pads and topical olive oil on episiotomy wound healing and showed no statistically significant difference between the CCGP and OO groups in terms of wound healing rates on the fifth and tenth day of delivery. The results also showed that the effectiveness of topical olive oil and cold gel pad on wound healing was the same [30]. In a clinical trial conducted by Kaviani et al., to determine the effect of olive leaf extract on episiotomy wound complications, the results showed a statistically significant difference in wound healing scores between the olive leaf ointment extract group and the other two groups on the third, seventh, tenth, and fourteenth day of the intervention (*P* < 0.05), [31]. In a study, Behmanesh et al. investigated the effect of sitting in an olive oil bath on the improvement of postpartum perineum injury. At the end of the study, a statistically significant difference was observed between the two groups in terms of the overall wound healing score (*P* < 0.001), and the use of olive oil, as an effective ingredient in episiotomy healing, was recommended [4].

### 4.6. Diaper Dermatitis

Diaper dermatitis is a common source of inflammation in neonates [55], and its prevalence has been reported to be up to 50 [56]. DR in the long term can damage the skin seriously leading to secondary infections and skin ulcers [57]. The long-term exposure to urine and feces may break down the skin integrity due to the presence of lipase and protease enzymes in urine [58].

Sharifi-Heris et al. conducted a study to compare the effect of olive and calendula ointments on diaper dermatitis. The results showed no statistically significant difference between the two groups in terms of the degree of inflammation before the intervention so that the degree of inflammation was 1.5% in both the olive group and the calendula group. Also, on the third, the fifth, and the seventh days after the intervention, the degree of inflammation did not show any significant difference. The results of this study showed that olives, similar to calendula, were effective in improving inflammation caused by diapers in children [7].

### 4.7. Atopic Dermatitis (Eczema)

In a study, Verallo-Rowell et al. investigated the antibacterial and emollient effects of coconut oil and virgin olive oil on atopic dermatitis. The two groups of VCO and VOO used olive oil and coconut oil in two noninfectious areas, then, *Staphylococcus aureus* was cultured in the areas, and the OSS-I score was obtained at the beginning and 4 weeks after the use of the oils. The results showed that after the intervention, only 1 (5%) person in the VCO group and 6 (50%) people in the VOO group were positive for *Staphylococcus aureus*. In terms of OSSI, there was a significant difference (*P*=0.004) between the two groups after the intervention, and the OSSI score decreased for both groups (*P*=0.005), but this decrease was greater in the VCO group [32]. In another study, Panahi et al. examined the effect of a cream containing olive oil and aloe vera on AD and compared it with topical betamethasone application. The results showed that the recovery rate in the olive group was 64.5% and in the betamethasone group was 13.5%, and a significant difference (*P* < 0.001) was observed between the two groups in that regard [33].

Squalene in olive oil has antioxidant and moisturizing properties [42] and can be used to treat people with seborrheic dermatitis, acne, psoriasis, and atopic dermatitis [59].

A wide range of studies has shown that olive oil is effective in pain relief and contains antioxidants that can slowly reduce the processes that cause pain in the body. Pure olive oil contains a natural chemical that acts as a painkiller. This substance, which is called oleocanthal [60], can have an analgesic effect by a mechanism similar to ibuprofen, which suppresses the production of prostaglandins [61–65], so it can relieve wound pain. It is important to note that several studies [4, 6, 27] have shown that olive oil has no side effects for consumers.

One of our limitations in this research was that we only searched and reviewed Persian and English articles and articles in other languages have not been reviewed. Another limitation was the lack of quality assessment of the studies. Therefore, the results of the studies should be interpreted with caution, and it is also possible that some related articles have not been reviewed in the present study, so it is recommended that more comprehensive research, especially systematic review articles, be conducted in this field.

According to the results of many studies, it seems that olive is a natural and safe substance that contains antioxidants, anti-inflammatory, antibacterial, and antiviral properties, and its use in wound healing or speeding up its process is recommended.

## 5. Conclusion

According to the above studies, olive and its products can be used by different methods to heal wounds and improve damage to the skin and mucous tissues or accelerate the wound healing process. The use of olive is safe, and access to it is easier and cheaper for most people with different education levels and cultures. It also, in addition to conventional treatments, increases the range of choice for people and minimizes the side effects of chemical treatments.

Today, with the advancement of technology, a variety of wound care techniques are expanding day by day, but there are still problems in pain management and delay or lack of wound healing, especially after surgery. New products can help by preventing barriers to wound healing, increasing wound healing stimuli, helping to accelerate wound healing, shortening the recovery period for definitive healing, and optimizing the end results. The production of wound healing products and methods and attention to all aspects of wound care can increase the ways to help patients with various types of wounds and can be effective in helping these patients.

## Figures and Tables

**Table 1:** Randomized trials evaluating the impact of topical application of olive on wound healing.

Author, year, and location	Study groups	Intervention	Variable measured/scale	Results
Nipple sore Oguz., 2014, Turkey [11]	OO (*n* = 56)	All patients: put OO on one nipple and lanolin on the other one and use the same ointment for the same nipple until the end of the study or use one of the modalities for both nipples if they were satisfied with the modality IP: 15 days	—	OO: fifty patients (89.2%) were more satisfied with OO L: 6 patients (10.8%) were more satisfied with L

Eshghizade., 2016, Iran [24]	OO (*n* = 30); AV (*n* = 30); HM (*n* = 30)	OO: 0.5 ml of OO 3 times a day AV: 0.5 ml of AV extract plus 3-4 drops of their milk 3 times a day HM: 3-4 drops of milk after each breastfeeding Place of use: the nipple and areola IP: 7 days	Storr scale	OO: 1.34 ± 0.55; AV: 1.00 ± 0.52; HM: 1.17 ± 0.53 (*P* < 0.06)

Pressure ulcer Varaei et al., 2019, Iran [8]	OO (*n* = 30); SAO (*n* = 30); C (*n* = 30); patients admitted to ICU	OO and SAO: both received a massage once a day with 1–3 mL of OO or SAO C: NI IP: 1 week Place of use: in the areas exposed to the risk of pressure ulcer	Braden's scale	OO: 11.76 ± 2.87; SAO: 12.20 ± 1.65; C: 11.86 ± 1.36 (*P* < 0.001)

Poursadra et al., 2019, Iran [25]	OO (*n* = 30); HA (*n* = 30); C (*n* = 30); patients admitted to ICU	OO: 15 ml of olive oil was gently applied once a day without any massage HA: a mixture of 1 g henna and 10 ml distilled water was applied for 30 min just once C: NI Place of use: PU grade one IP: 7 days	Pressure ulcer scale for healing (PUSH) tool	OO: 5.44 ± 3.806; HO: 3.39 ± 3.54; C: 9.83 ± 2.864 (*P* < 0.001); the mean area of the ulcer on days 4 and 7 in the HO was lower than that in the OO and control groups (*P* < 0.001)

Miraj et al., 2020, Iran [26]	OO (*n* = 36); C (*n* = 36); patients admitted to ICU	OO: 15 ml OO was rubbed gently on the wounded area once a day for 30 min without massage; the area was washed with tepid water and the skin was dried Place of use: PU grade one C: NI IP: 7 days	Pressure ulcer scale for healing (PUSH) tool	OO: 5.44 ± 3.806; C: 8.83 ± 2.864 (*P* < 0.001)

Díaz-Valenzuela et al., 2019, Spain [27]	HOFA (*n* = 274); OO (*n* = 263)	OO and HOFA: solutions (2 sprays; one spray delivers 0.2 mL) were applied on at-risk skin areas every 12 hours IP: 30 days or until pressure ulcer onset Place of use: at-risk skin areas	PU incidence	PU incidence OO: 4.18%; HOFA: 6.57%; PU incidence difference: −2.39% (95% CI = −6.40 to 1.56%):

Bajwa et al., 2017, Spain [28]	OO (*n* = 30) C (*n* = 30)	OO: topical 15 cc premium and standard formula olive oil once a day C: NI Place of use: at-risk areas of patient bodies without any massaging IP: 3 weeks	Bedsore incidence	OO: 5 patients (16%) had developed bedsore after an average of 18.73 ± 5.36 days C: 12 patients (40%) had developed bedsore after an average of 15.46 ± 7.40 days (*P*=0.03)

Chronic ulcers Vitsos et al., 2019, Greece [9]	*Ceratothoa oestroides* OO (*n* = 40); pilot study	Ointment was applied once daily IP: 3 months	Bates–Jensen wound assessment tool (BWAT)	*C. oestroides* decreased of 36% in score of ulcers (*P* < 0.001); the decrease being significant from the first month (*P* < 0.007)

Diabetic wounds Nasiri et al., 2015, Iran [6]	OO (*n* = 17); C (*n* = 17)	OO: applied olive oil topically to the wound area once a day for four weeks C : NI	Wagner system	OO: 391.33 ± 15.05; C: 348.00 ± 43.08 (*P*=0.001)

Karimi et al., 2015, Iran [29]	In total, the research units were 45 people and were divided into three groups: OO, H, and C )the exact number of each group is not mentioned(	OO: once a day, the wound was covered with gauze soaked in olive oil (4 cc) H: they used honey once a day on the wound C: NI IP: 1 month	Wagner system	The mean score of wound healing was OO: 371.5; H: 330.5; C: 268.0; (*P*=0.002)

Incisional wounds (perineal ulcer) Amani et al., 2015, Iran [30]	OO (*n* = 45); CCGP (*n* = 45)	OO: twice a day, first for 12 hours after delivery and then for 10 days after delivery CCGP: for 20 minutes, first for 12 hours after delivery and then up to 10 days if necessary	REEDA scale	OO: 0/2 ± 0/5; CCGP: 0/47 ± 0/97 (*P*=0.01)

Kaviani et al., 2019, Iran [31]	Olive leaf (*n* = 30); placebo (*n* = 30); C: (*n* = 30)	Olive leaf: olive leaf extract ointment was used (3 times a day for 10 days); Placebo: placebo was used (3 times a day for 10 days); C: betadine solution was used (3 times a day for 10 days)	REEDA scale	Olive leaf: 0; placebo: 0.43 ± 0.56; C: 0.63 ± 0.76; (*P* < 0.001)

Behmanesh et al., 2012, Iran [4]	OO (*n* = 30); C (*n* = 30)	OO: for the first time, 24 hours after delivery and then for ten days after delivery, they used a sitting bath of olive oil (10 drops of OO in 5 liters of water) for 10 minutes twice a day; C: in the same way but they used distilled water instead of OO	REEDA scale	OO: 0/30 ± 0/46; C: 2/10 ± 1/77 (*P* < 0.001)

Diaper dermatitis (contact dermatitis) Sharifi‐Heris et al., 2018, Iran [7]	Olive ointment 1.5% (*n* = 37); calendula ointment 1.5% (*n* = 39)	Olive and calendula: both were treated with the respective topical after diaper changing per day IP: 7 days	Scale point six	There is no significant difference between the two groups on the third (*P*=0.413), fifth (*P*=0.17), and seventh (*P* > 0.999)

Atopic dermatitis (eczema) Verallo-Rowell et al., 2008, Philippines [32]	VCO (*n* = 26); VOO (*n* = 26)	VCO and VOO: 5 cc twice daily at two noninfected sites IP: 4 weeks	SCORAD severity index of atopic dermatitis (O–SSI) scoring	VCO: 22.6 ± 3.6; VOO: 26.7 ± 5.7 (*P* > 0.004)

Panahi et al., 2020, Iran [33]	Olivederma (*n* = 19); betamethasone (*n* = 16); AD patients	Olivederma: received topical olivederma 2 times a day Betamethasone: received topical betamethasone 2 times a day IP: 6 weeks	SCORAD severity index of atopic dermatitis (O–SSI) scoring	Olivederma: 50.5 ± 10.7 Betamethasone: 23.9 ± 13.3 (*P* < 0.001) Percentage of recovery Olivederma: 64.5% Betamethasone: 13.5% (*P* < 0.001)

Psoriasis Acosta et al., 2016, Spain [34]	Alyvium (*n* = 15); placebo (*n* = 15)	Alyvium: two capsules (which contains 500 mg of an olive polyphenolic extract, 200 *μ*g vitamin A, 0.35 mg riboflavin, and 12.5 *μ*g of biotin per capsule) a day Placebo: two capsules a day of maltodextrins IP: 12 weeks	Psoriasis area and severity index; PASI	Alyvium: 3.0 ± 1.47; Placebo: 3.27 ± 2.57 (*P* < 0.05)

OO: olive oil; PU: pressure ulcer; HA: henna oil; CCGP: cold compress with gel pack; ARR: absolute risk reduction; MD: mean difference; HOFA: hyperoxygenated fatty acids; IP: intervention period; VOO: virgin olive oil; VCO: virgin coconut oil; AE: atopic eczema; SCORAD: SCORing atopic dermatitis; SA: sweet almond; HM: human milk; AV: aloe vera; H: honey; NI: no intervention; GA: gestational age; C: control; DR: diaper dermatitis.

## Abbreviations

PRISMA: Preferred Reporting Items For Systematic Reviews and Meta-Analyses
CINAHL: Cumulative index to nursing and allied health literature
SID: Scientific information database
CNLO: Congenital nasolacrimal duct obstruction
OO: Olive oil
OOPCs: Olive oil phenolic compounds
EVOO: Extra virgin olive oil
PU: Pressure ulcer
SAO: Sweet almond
PUSH: Pressure ulcer scale for healing
HO: Henna oil
EPUAP: European pressure ulcer advisory panel
DFU: Diabetic foot ulcer
DM: Diabetes mellitus
AV: Aloe vera
HM: Human milk
IP: Intervention period
NI: No intervention
C: Control
CI: Confidence interval
ARR: Absolute risk reduction
MD: Mean difference
HOFA: Hyperoxygenated fatty acid
VOO: Virgin olive oil
VCO: Virgin coconut oil
SCORAD: Severity index of atopic dermatitis (O–SSI) scoring; atopic dermatitis
SA: Sweet almond
GA: Gestational age
H: Honey
NI: No intervention
BWAT: Bates-Jensen wound assessment tool
CCGP: Cold compress with gel pack
ARR: Absolute risk reduction
AD: Atopic dermatitis.
